# The prevalence of stroke and depression and factors associated with depression in elderly people with and without stroke

**DOI:** 10.1186/s12877-016-0347-6

**Published:** 2016-10-07

**Authors:** Carl Hörnsten, Hugo Lövheim, Peter Nordström, Yngve Gustafson

**Affiliations:** Department of Community Medicine and Rehabilitation, Geriatric Medicine, Umeå University, SE-90185 Umeå, Sweden

**Keywords:** Stroke, Depression, Epidemiology

## Abstract

**Background:**

Few studies have investigated factors associated with depression among elderly people with and without stroke concurrently, using identical settings, procedures and study variables. The aim was to investigate the prevalence of stroke and depression and to compare the factors associated with depression in people with and without stroke.

**Methods:**

A postal mail survey was sent to 65-, 70-, 75- and 80-year-olds in northern Sweden and Finland in 2010 (*n* = 6098). Stroke was defined as answering “yes” to the question “Have you had a stroke?” Depression was defined as answering “yes” to the question “Are you depressed?” or having a Geriatric Depression Scale-4 score ≥2. Dependence in personal activities of daily living was defined as not showering without human assistance. Associations were tested with log-binomial regression.

**Results:**

The overall stroke prevalence was 7.0 ± 0.3 % and increased from 4.7 ± 0.4 % among 65-year-olds to 11.6 ± 1.0 % among 80-year-olds (*p* < 0.001). The overall depression prevalence was 12.8 ± 0.4 % and increased from 11.0 ± 0.6 % among 65-year-olds to 18.1 ± 1.2 % among 80-year-olds (*p* < 0.001). Depression was more common among people with stroke (Prevalence Ratio 1.77, 95 % Confidence Interval 1.48-2.12).

In the non-stroke group, depression was independently associated with diabetes, dependence in instrumental activities of daily living, living alone, not having someone to talk to, poor finances, pain problems and having a life crisis in the preceding year. In the group with stroke, depression was independently associated with dependence in personal activities of daily living and having a life crisis the preceding year.

**Conclusions:**

Depression in people without stroke appeared to be independently associated with a broader range of external factors than depression in people with stroke.

**Electronic supplementary material:**

The online version of this article (doi:10.1186/s12877-016-0347-6) contains supplementary material, which is available to authorized users.

## Background

People who have had a stroke have a higher prevalence of depression than those who have not, both cross-sectionally [1, 2] and after a set interval after suffering a stroke [3, 4]. While it is an established notion that suffering a stroke can lead to depression, some studies are also suggesting that depression may lead to an increased incidence of stroke [5, 6]. Depression in people with stroke has been linked to increased mortality [7, 8], and reduced physical functioning [9, 10] and well-being [11]. Both the prevalence [12, 13] and incidence [14] of stroke rise notably with age, which is also true for the prevalence [15] and incidence [16] of depression.

In people with stroke, depression has been consistently associated with stroke severity, functional disability and cognitive impairment [17]. It has been proposed that the brain damage incurred after a stroke is an important causative factor for depression [18]. This suggestion is supported by the association between ischemic lesions and behavioral symptoms [18], by the association between stroke and depression [3, 4] and also by the association between stroke severity and depression [17, 19]. Regarding elderly populations in general, depression has been consistently associated with bereavement, sleep disturbance, functional disability, prior depression and female sex [20].

While the characteristics, diseases and functional abilities associated with depression have been investigated in multiple elderly populations [20] and stroke disease cohorts [17], few studies have investigated factors associated with depression among elderly people with and without stroke concurrently, using identical settings, procedures and study variables. Such a study would make it possible to investigate if the impact of social factors, demographics, comorbidity and functional ability on depression risk differ between elderly people with stroke compared with elderly people without stroke. Methodological differences between studies make it difficult to achieve this comparison based on separate studies of people with and without stroke.

The aim of this paper was to describe the prevalence of stroke and depression in 65-, 70-, 75- and 80-year-olds in northern Sweden and Finland, and to investigate the factors associated with depression among elderly people who have had stroke compared to those without stroke.

## Methods

### Setting

Data from the Gerontological Regional Database (GERDA) survey were used. The survey targeted the 15 municipalities in the county of Västerbotten in Sweden and the 17 municipalities in the county of Österbotten in Finland. The survey was sent out to 65-, 70-, 75- and 80-year-olds in 2010. All individuals in their respective age groups were contacted in the rural municipalities, but only a randomly selected third of potential participants were contacted in the two urban municipalities in Sweden (Umeå, Skellefteå) and half in the urban municipality in Finland (Vaasa). The Regional Ethical Review Board of Umeå/Sweden approved the study (2010-220-32Ö).

### Participants

The survey was sent to 5425 people in Sweden and 5271 in Finland, of whom 3779 (69.7 %) and 3059 (58.0 %) respectively responded, resulting in an overall response rate of 6838 out of a possible 10696 (63.9 %). Of those who responded, the 6098 (89.2 %) who answered the questions about having had a stroke and being depressed or the four relevant questions from a depression assessment scale were selected for the final sample. The final sample included 2441 of the 3925 (62.2 %) 65-year-olds, 1496 of the 2507 (59.7 %) 70-year-olds, 1246 of the 2310 (53.9 %) 75-year-olds and 915 of the 1954 (46.8 %) 80-year-olds, to whom the survey was sent.

### Procedure

The postal mail survey was posted in October 2010 in Sweden and Finland. It included 75 items, covering demographic information and interests, and questions about health, attitudes and disabilities. One reminder letter was sent to participants who did not respond.

### Stroke definition

In the present study, having a previous stroke was defined as answering “yes” to the question “Have you had a stroke?”. In a German demographic cohort of people aged 65 years and older [21], a single stroke question resulted in a sensitivity of 65.8 against registered diagnoses in medical records and neurological examinations in uncertain/equivocal cases. The single stroke question resulted in more false negatives compared with a concurrently tested 6-item stroke assessment scale, but also had notably fewer false positives [21].

### Depression definition

The Geriatric Depression Scale (GDS) 4-item version [22] is a short assessment for depression comprising four yes/no questions. The four questions are drawn from the widely used GDS 15-item assessment scale [23]. Multiple 4-item versions of the GDS exist, depending on which of the 15 questions of the GDS-15 that are being used. The version used in this paper consisted of the questions “Are you basically satisfied with your life?”, “Do you feel that life is empty?”, “Are you afraid that something bad is going to happen?” and “Do you feel happy most of the time?”. In a primary care setting, with a cut-off at two or more points, the GDS-4 was found to have a sensitivity of 61 % and a specificity of 81 % measured against a comprehensive diagnostic interview [22].

The present study also included the yes/no question “Do you feel depressed?”. In a stroke disease cohort [24], the question “Do you often feel sad or depressed?” from the Yale-Brown Obsessive compulsive scale [25] resulted in a sensitivity of 86 % and a specificity of 78 % against a ≥6 cutoff on the Montgomery Åsberg Depression Rating Scale.

Depression was defined as answering “yes” to the yes/no question, or a GDS-4 score ≥2. This produced an overall figure of 739 people with depression; 290 people selected on the result of the GDS-4 cutoff alone, 294 people on the yes/no question alone, and 155 people who met both criteria. A combination measure was selected to increase the sensitivity for depression.

### Other definitions

Malignancy, diabetes and a previous myocardial infarction were defined as answering “yes” to yes/no questions.

Dependence in personal activities of daily living (ADL) was defined as answering “no” to the question “Do you shower without human assistance?”. Dependence in instrumental ADL was defined as answering “no” to any of the following questions: “Do you clean your residence by yourself, without human assistance?”, “Do you buy your own groceries without human assistance?”, “Do you utilize public transportation without human assistance?” and “Do you cook your own food without human assistance?”. Vision impairment was defined as answering “no” to the question “Are you able to read the text in a newspaper?”. Hearing impairment was defined as answering “no” to the question “Are you able to hear what someone tells you in a normal tone of voice from a distance of about 1 meter?”. Weight loss was defined as answering “yes” to the question “Has your weight decreased in the last three months?”.

Living alone was defined as answering “I live alone” or “I am living with someone, but not in the same residence” to the question “Do you live together with someone?”. Having no one to talk to was defined as answering “no” to the question “Do you have someone you can talk to about anything, someone you can share your concerns as well as your joys with?”.

Having financial difficulties was defined as answering “It is pretty difficult” or “It is very difficult”, but not “With some difficulty” or “Without any difficulty”, to the question “Do you make ends meet?”. Having a pain problem was defined as answering “yes” to the question “Have you had any aches or pain in the last week?”. Having a current life crisis was defined as answering “yes” to the question “Have you experienced something that you would describe as a life crisis the last year (12 months)?” A comprehensive list of the questions and answers that the present study is based on is provided as Additional files 1 and 2.

### Statistics

R version 3.0.2 was used for statistical analyses. The prevalence of stroke and depression and the corresponding prevalence ratio in the population were estimated using an R software library for complex surveys called “Survey”. These epidemiological results were weighted based on the proportion of participants compared with known population totals in demographic strata based on home municipality, sex and age and, adjusted for finite population correction. Differences in prevalence between age groups, men and women, and Sweden and Finland were tested using ChiSquare tests, and the prevalence ratios between stroke and depression were tested using log-binomial regression models. Log-binomial regression models were used instead of the more widespread logistic regression models in order to decrease the likelihood of distorted rate ratios.

Factors associated with depression were also tested using log-binomial regression models. These analyses were not adjusted for the composition of the underlying population. Out of 75 items in the survey, 18 that had been associated with depression in previous studies [17, 20] or were thought to be relevant based on clinical experience were entered into univariate models. Separate models were used for people with and without stroke.

The influence of stroke status on the univariate association between depression and the study items was explicitly tested using interaction analyses. Likelihood-ratio tests were used to compare interaction models of the form “item + stroke status + item * stroke status (interaction term)” with the corresponding models without interaction terms.

Factors significantly associated with depression in univariate models, and age and sex, were entered into two multivariate models: one for those with and one for those without stroke. Multivariate models were analyzed according to a previously published methodology [26], utilizing a restricted optimization routine to increase the probability of regression convergence. A value of *p* < 0.05 was considered statistically significant.

## Results

In the final sample, 425 of 6098 (7.0 %) people had a stroke and 739 (12.1 %) had depression. Adjusted for the composition of the population, the stroke prevalence was 7.0 ± 0.3 % overall, and increased from 4.7 ± 0.4 % among the 65-year-olds to 11.6 ± 1.0 % among the 80-year-olds (*p* < 0.001). The adjusted stroke prevalence was higher among men, 8.4 ± 0.5 % compared with 5.7 ± 0.4 % for women (*p* < 0.001), and higher in Sweden, 7.9 ± 0.4 % compared with 5.6 ± 0.4 % in Finland (p < 0.001). The adjusted depression prevalence was 12.8 ± 0.4 % overall, and increased from 11.0 ± 0.6 % among the 65-year-olds to 18.1 ± 1.2 % among the 80-year-olds (*p* < 0.001). The adjusted depression prevalence was 13.5 ± 0.6 % among women compared with 12.0 ± 0.6 % for men (p = 0.074), and 12.9 ± 0.6 % in Sweden compared with 12.8 ± 0.6 % in Finland (*p* = 0.950). Depression was 1.77 (95 % CI 1.48–2.12) times more common among those with stroke and this association was consistent among women, men, Swedish people, Finnish people, 65-year-olds, 70-year-olds and 75-year-olds, but the association did not reach significance among 80-year-olds (Table 1).

**Table 1:** The prevalence of stroke and depression and the corresponding prevalence ratio in total and in demographic sub-groups

	Stroke % ± SE	Depressed % ± SE	PR (CI)
Total, *n* = 6098	7.0 ± 0.3	12.8 ± 0.4	1.77 (1.48–2.12)
65 years, *n* = 2441	4.7 ± 0.4	11.0 ± 0.6	1.91 (1.38–2.64)
70 years, *n* = 1496	5.8 ± 0.5	11.1 ± 0.8	1.74 (1.14–2.64)
75 years, *n* = 1246	8.5 ± 0.7	13.5 ± 0.9	1.80 (1.26–2.58)
80 years, *n* = 915	11.6 ± 1.0	18.1 ± 1.2	1.36 (0.96–1.92)
Men, *n* = 2831	8.4 ± 0.5	12.0 ± 0.6	1.59 (1.22–2.07)
Women, *n* = 3267	5.7 ± 0.4	13.5 ± 0.6	2.03 (1.59–2.58)
Sweden, *n* = 3465	7.9 ± 0.4	12.9 ± 0.6	1.63 (1.28–2.07)
Finland, *n* = 2633	5.6 ± 0.4	12.8 ± 0.6	2.07 (1.59–2.70)

*SE* standard error, *PR* prevalence ratio, *CI* confidence interval. The results were weighted based on the proportion of participants compared with known population totals in demographic strata based on home municipality, sex and age and, adjusted for finite population correction. Prevalence ratios were calculated using univariate log-binomial regression analyses

The sample was divided into two groups, based on stroke status, for univariate analyses of factors associated with depression, as shown in Table 2. In the non-stroke group, depression was associated with urban dwellers, myocardial infarction, cancer, diabetes, dependence in personal and instrumental ADL, impaired vision, impaired hearing, weight loss, living alone, not having someone to talk to, poor finances, pain problems, a life crisis in the preceding year and increasing age. In the group with stroke, depression was associated with dependence in personal ADL, impaired vision, living alone, not having someone to talk to, poor finances, pain problems and a life crisis in the preceding year. The associations of depression with cancer (*p* = 0.026), not having someone to talk to (*p* = 0.013) and poor finances (*p* = 0.029) were weaker in people who had had a stroke than in people without stroke according to interaction analyses of the whole sample.

**Table 2:** Factors associated with depression in people with and without stroke

	No stroke			Stroke		
	No depression *n* = 5028 (%)	Depression *n* = 645 (%)	PR (95 % CI)	No depression *n* = 331 (%)	Depression *n* = 94 (%)	PR (95 % CI)
Women	2716 (54.0)	361 (56.0)	1.07 (0.93–1.24)	142 (42.9)	48 (51.1)	1.29 (0.90–1.85)
Finnish	2210 (44.0)	288 (44.7)	1.03 (0.89–1.19)	99 (29.9)	36 (38.3)	1.33 (0.92–1.90)
Urban	1539 (30.6)	235 (36.4)	1.26 (1.08–1.46)	94 (28.4)	25 (26.6)	0.93 (0.61–1.37)
MI	297 (6.1)	56 (9.0)	1.44 (1.11–1.83)	64 (24.0)	22 (27.8)	1.17 (0.74–1.76)
Cancer	627 (12.7)	108 (17.2)	1.37 (1.12–1.65)	65 (22.0)	14 (16.7)	0.76 (0.43–1.23)^a^
Diabetes	548 (11.2)	108 (17.6)	1.59 (1.30–1.91)	46 (16.0)	19 (24.1)	1.47 (0.92–2.23)
Dep. P-ADL	248 (5.0)	59 (9.2)	1.76 (1.37–2.22)	34 (10.3)	26 (28.0)	2.35 (1.60–3.32)
Dep. I-ADL	1392 (28.8)	268 (43.2)	1.74 (1.50–2.01)	173 (54.2)	57 (62.6)	1.31 (0.91–1.94)
Imp. vision	80 (1.6)	22 (3.4)	1.93 (1.28–2.73)	11 (3.3)	8 (8.6)	2.00 (1.02–3.21)
Imp. hearing	59 (1.2)	14 (2.2)	1.71 (1.00–2.62)	7 (2.1)	1 (1.1)	0.56 (0.03–2.05)
Weight loss	246 (4.9)	48 (7.6)	1.48 (1.11–1.91)	24 (7.3)	8 (8.8)	1.17 (0.56–2.02)
Lives alone	1175 (23.6)	255 (39.9)	1.95 (1.68–2.25)	92 (28.3)	37 (39.4)	1.46 (1.01–2.08)
No one to talk to	86 (1.7)	58 (9.1)	3.79 (3.02–4.64)	12 (3.7)	9 (9.6)	2.01 (1.07–3.16)^a^
University ed.	744 (15.1)	84 (13.3)	0.87 (0.70–1.08)	38 (11.8)	11 (12.0)	1.01 (0.54–1.67)
Poor finances	224 (4.7)	96 (15.5)	2.89 (2.38–3.46)	30 (9.7)	16 (18.6)	1.74 (1.06–2.63)^a^
Pain problem	3207 (65.0)	487 (77.2)	1.71 (1.44–2.05)	227 (70.5)	75 (81.5)	1.64 (1.04–2.75)
Life crisis	1949 (39.6)	402 (63.1)	2.33 (2.01–2.72)	168 (51.5)	62 (66.7)	1.64 (1.13–2.45)
Age	70.3 ± 5.4	71.3 ± 5.8	1.03 (1.02–1.04)	72.3 ± 5.7	72.7 ± 5.9	1.01 (0.98–1.04)

*PR* prevalence ratio, *CI* confidence interval, *MI* myocardial infarction, *Dep. P-ADL* dependence in personal activities of daily living, *Dep. I-ADL*, dependence in instrumental activities of daily living, *Imp. vision* impaired vision, *Imp. hearing* impaired hearing, *University ed*. University educated. Denominators for proportions may vary due to missing values. Associations were estimated as prevalence ratios, using univariate log-binomial regression analyses

^a^Having had a stroke significantly affected the association between depression and the variable in an interaction analysis performed on the whole sample

The variables sex, age and factors significantly associated with depression in the univariate analyses were entered into separate multivariate analyses for the groups with and without stroke, as shown in Table 3. In the non-stroke group, depression was independently associated with diabetes (PR 1.26, CI 1.04–1.48), dependence in instrumental ADL (PR 1.53, CI 1.32–1.73), living alone (PR 1.78, CI 1.57–2.00), not having someone to talk to (PR 1.96, CI 1.59–2.33), poor finances (PR 1.91, CI 1.63–2.20), pain problems (PR 1.55, CI 1.31–1.80) and a life crisis the preceding year (PR 1.79, CI 1.57–2.01). Due to the close relationship between personal and instrumental ADL, the multivariate model for the non-stroke group was repeated for all variables except instrumental ADL, but depression was still not independently associated with dependence in personal ADL (data not shown). In the group with stroke, depression was independently associated with dependence in personal ADL (PR 2.02, CI 1.46–2.58) and a life crisis the preceding year (PR 1.53, CI 1.02–2.05).

**Table 3:** Factors independently associated with depression in people with and without stroke

	No stroke	Stroke
	PR (95 % CI)	PR (95 % CI)
Women	0.99 (0.83–1.15)	1.16 (0.75–1.56)
Age	1.01 (0.99–1.02)	1.01 (0.98–1.04)
Urban	1.09 (0.93–1.26)	–
MI	1.10 (0.80–1.40)	–
Cancer	1.12 (0.90–1.33)	–
Diabetes	1.26 (1.04–1.48)	–
Dep. P-ADL	0.85 (0.53–1.16)	2.02 (1.46–2.58)
Dep. I-ADL	1.53 (1.32–1.73)	–
Imp. vision	1.39 (0.86–1.91)	1.48 (0.59–2.38)
Imp. hearing	1.07 (0.47–1.66)	–
Weight loss	0.99 (0.66–1.31)	–
Lives alone	1.78 (1.57–2.00)	1.31 (0.87–1.75)
No one to talk	1.96 (1.59–2.33)	1.00 (0.27–1.73)
Poor finances	1.91 (1.63–2.20)	1.31 (0.72–1.89)
Pain problem	1.55 (1.31–1.80)	1.56 (0.94–2.18)
Life crisis	1.79 (1.57–2.01)	1.53 (1.02–2.05)

*PR* prevalence ratio, *CI* confidence interval, *MI* myocardial infarction, *Dep. P-ADL* dependence in personal activities of daily living, *Dep. I-ADL* dependence in instrumental activities of daily living, *Imp. vision* impaired vision, *Imp. hearing* impaired hearing. Associations were estimated as prevalence ratios, using multivariate log-binomial regression analyses

## Discussion

The prevalence of stroke and depression increased with increasing age. Stroke was more common among men than women, and more common in Sweden than in Finland. Stroke was associated with depression overall, among men, women, Swedish people and Finnish people. In people with stroke, depression was independently associated with dependence in personal ADL and a life crisis the preceding year. In people without stroke, depression was not associated with dependence in personal ADL, but was independently associated with a larger number of external factors.

The age-specific prevalence of stroke appeared to increase with increasing age. Our estimated stroke prevalence figures are in line with previously published self-reported results [12, 13]. The higher stroke prevalence found for men than for women is in line with previous studies [12, 13]. The age-specific prevalence of depression also appeared to increase with increasing age. Our estimated depression prevalence figures are in line with previous pooled estimates of the prevalence of all depressive disorders, although this estimate varies greatly depending on how depression is defined [15].

Being depressed was 1.77 times more common among people with a previous stroke in the full sample, which remained true in all age, sex and country sub-groups, but did not reach significance in 80-year-olds. It is possible that lower power due to fewer participants and a lower participation rate among the 80-year-olds contributed to the non-significant result, but on the other hand both the prevalence of stroke and depression were higher among 80-year-olds than other age groups. It also has to be considered that the factors associated with depression may differ among 80-year-olds from those in elderly people in lower age groups, which could explain our result. Previous studies have found people who have had a stroke to have a higher prevalence of depression than those who have not, both cross-sectionally [1, 2] and after a set interval after suffering a stroke [3, 4].

The association between dependence in personal ADL and depression in people with stroke is in line with previously published results [17]. The association between having a life crisis and depression in people with stroke has not been widely investigated, but social distress was found to be associated with depression in people with stroke in a previous study [27]. Regarding people without stroke, the association between instrumental ADL and depression is in line with disability being associated with depression in a meta-analysis of community dwellers [20], although personal ADL was not independently associated with depression in people without stroke in the present study. A life crisis in the preceding year was also associated with depression in people without stroke, which is in line with previously reported associations between bereavement and depression among community-dwellers [20].

Depression was associated with a larger number of external factors in people without stroke, such as living alone, not having someone to talk to and poor finances, but also diabetes, and having a pain problem. The difference regarding external factors was further supported by interaction analyses of the whole sample, where having had a stroke appeared to weaken the associations between depression and having someone to talk to, poor finances and cancer.

It is possible that among people with stroke, debilitating physical disability denoted by dependence in personal ADL is associated with depression, while external factors are less relevant. One possible explanation is that dependence in personal ADL is likely to be linked to stroke severity, or the extent of brain tissue damage after stroke, which could be a particularly important causative factor for depression in stroke survivors. In support of this, previous studies have shown a clear association between stroke severity and depression [17, 19] and also between ischemic lesions and behavioral symptoms [18]. The underlying biological mechanism may be decreased amine levels due to brain tissue damage [28], and/or possibly inflammation in response to perfusion deficits [29].

However, it is also possible that people with stroke may be particularly sensitive to the social consequences of loss of independence in personal ADL. In the already vulnerable state of having suffered a stroke, dependence in personal ADL may induce feelings of helplessness, reduce self-esteem and ultimately be a pathway to depression. Among people without stroke, external conditions such as their financial situation, living alone and not having someone to confide in, appeared to be more important as factors associated with depression.

The data in the present study is not sufficient to provide grounds for differentiated treatment of depression between people with and without stroke, however the results may indicate that different approaches are needed for treatment and prevention of depression in people with and without stroke. The different characteristics of and risk factors for depression in people with and without stroke should be the subject of further research.

### Strengths and limitations

Stroke prevalence was determined by self-reporting using a yes/no question. While some mischaracterized cases are to be expected when using self-reported data, our age-specific stroke prevalence estimates are similar to previous self-reported results [12, 13], and only slightly higher than self-reported cases confirmed by medical documentation [12]. One benefit of using self-reported data is that it made it possible to reach a large number of people representing both urban and rural community-dwellers.

Depression prevalence was determined by a combination of self-reporting with a yes/no question and the use of a depression assessment scale. While depression assessment with a yes/no question has been used successfully in stroke patients [24], depression is known to be underdiagnosed in elderly people [30], meaning that patients who are not diagnosed with depression and do not consider themselves depressed may be overlooked. In addition, our clinical experience suggests that elderly people who are depressed may not be open about it when asked a direct question. We decided to combine self-reporting with a short version of a depression assessment scale to increase the sensitivity for depression. Our estimated depression prevalence was similar to the pooled prevalence of depressive disorders reported in a recent meta-analysis [15].

The investigation of factors associated with depression was limited to items suitable to a postal survey. This precluded the use of any radiological assessment of stroke severity, blood tests or review of medical charts. There was no assessment of stroke severity at the time when the participant had his/her stroke, although long-term functional outcome was assessed with survey items for ADL dependency.

Associations with depression were calculated using log-binomial regression, making it possible to present prevalence ratios. These are easier to interpret than the widely used odds ratios as they are not distorted when analyzing prevalent outcomes. One possible negative aspect of this choice is that it is more difficult to compare with previously published cross-sectional results, as they mainly report associations as odds ratios. Using log-binomial regression is also difficult in most statistical software because of non-convergent optimization procedures in multivariate analyses, and the method used here, involving constrained optimization, is not widely utilized.

In the analytical part of the study, the rural areas were oversampled compared to the urban areas, which might have influenced the results. While survey weights were available, they were used only for the estimates of stroke and depression prevalence that make up the epidemiological part of the study. There is a scientific consensus that descriptive results should be weighted if the survey sampling was unbalanced, but there is no such consensus regarding analytical results [31]. The response rate in the present study was, however, fairly high, including a substantial number of people living both in urban and rural settings.

## Conclusions

In the present study, the prevalence of stroke and depression rose with increasing age. Stroke appeared to be more common among men. Depression in people without stroke seemed to be independently associated with a broader range of external factors, while depression in people with stroke was only associated with dependence in personal ADL and having had a life crisis the preceding year.

## Additional files

Additional file 1: Survey questions. Contains the questions and answers that the present study is based on. (PDF 15 kb)
Additional file 2: Study data. Contains the data that the present study is based on as a csv file. (CSV 287 kb)

## Abbreviations

ADL: Activities of daily living
CI: Confidence interval
GDS: Geriatric depression scale
GERDA: Gerontological regional database
PR: Prevalence ratio

## References

[1] Beekman AT, Penninx BW, Deeg DJ (1998). Depression in survivor of stroke: A community-based study of prevalence, risk factors and consequences. Soc Psychiatry Psychiatr Epidemiol..

[2] Hornsten C, Molander L, Gustafson Y (2012). The prevalence of stroke and the association between stroke and depression among a very old population. Arch Gerontol Geriatr..

[3] Kase CS, Wolf PA, Kelly-Hayes M (1998). Intellectual decline after stroke: The Framingham study. Stroke..

[4] Linden T, Blomstrand C, Skoog I (2007). Depressive disorders after 20 months in elderly stroke patients: A case-control study. Stroke..

[5] Liebetrau M, Steen B, Skoog I (2008). Depression as a risk factor for the incidence of first-ever stroke in 85-year-olds. Stroke..

[6] Salaycik KJ, Kelly-Hayes M, Beiser A (2007). Depressive symptoms and risk of stroke: The Framingham study. Stroke.

[7] Hornsten C, Lovheim H, Gustafson Y (2013). The association between stroke, depression, and 5-year mortality among very old people. Stroke..

[8] Morris PL, Robinson RG, Andrzejewski P (1993). Association of depression with 10-year poststroke mortality. Am J Psychiatry..

[9] Alghwiri AA (2016). The correlation between depression, balance, and physical functioning post stroke. J Stroke Cerebrovasc Dis..

[10] Hadidi N, Treat-Jacobson DJ, Lindquist R (2009). Poststroke depression and functional outcome: A critical review of literature. Heart Lung..

[11] Lofgren B, Gustafson Y, Nyberg L (1999). Psychological well-being 3 years after severe stroke. Stroke..

[12] Bots ML, Looman SJ, Koudstaal PJ (1996). Prevalence of stroke in the general population. The Rotterdam study. Stroke.

[13] Jungehulsing GJ, Muller-Nordhorn J, Nolte CH (2008). Prevalence of stroke and stroke symptoms: A population-based survey of 28,090 participants. Neuroepidemiology..

[14] Rothwell PM, Coull AJ, Giles MF (2004). Change in stroke incidence, mortality, case-fatality, severity, and risk factors in Oxfordshire, UK from 1981 to 2004 (Oxford vascular study). Lancet..

[15] Luppa M, Sikorski C, Luck T (2012). Age- and gender-specific prevalence of depression in latest-life – systematic review and meta-analysis. J Affect Disord..

[16] Palsson SP, Ostling S, Skoog I (2001). The incidence of first-onset depression in a population followed from the age of 70 to 85. Psychol Med..

[17] Hackett ML, Anderson CS (2005). Predictors of depression after stroke: A systematic review of observational studies. Stroke..

[18] Alexopoulos GS, Meyers BS, Young RC (1997). 'Vascular depression' hypothesis. Arch Gen Psychiatry..

[19] Appelros P, Viitanen M (2004). Prevalence and predictors of depression at one year in a Swedish population-based cohort with first-ever stroke. J Stroke Cerebrovasc Dis..

[20] Cole MG, Dendukuri N (2003). Risk factors for depression among elderly community subjects: A systematic review and meta-analysis. Am J Psychiatry..

[21] Berger K, Hense HW, Rothdach A (2000). A single question about prior stroke versus a stroke questionnaire to assess stroke prevalence in populations. Neuroepidemiology..

[22] D'Ath P, Katona P, Mullan E (1994). Screening, detection and management of depression in elderly primary care attenders. I: The acceptability and performance of the 15 item Geriatric Depression Scale (GDS15) and the development of short versions. Fam Pract.

[23] Sheikh JI, Yesavage JA, Brink TL (1986). Geriatric Depression Scale (GDS): Recent evidence and development of a shorter version. Clinical gerontology: A guide to assessment and intervention.

[24] Watkins C, Daniels L, Jack C (2001). Accuracy of a single question in screening for depression in a cohort of patients after stroke: Comparative study. BMJ..

[25] Goodman WK, Price LH, Rasmussen SA (1989). The Yale-Brown Obsessive Compulsive Scale. I. Development, use, and reliability. Arch Gen Psychiatry.

[26] Borba de Andrade B, Carabin H (2011). On the estimation of relative risks via log binomial regression. Rev Bras Biom.

[27] Andersen G, Vestergaard K, Ingemann-Nielsen M (1995). Risk factors for post-stroke depression. Acta Psychiatr Scand..

[28] Loubinoux I, Kronenberg G, Endres M, Schumann-Bard P, Freret T, Filipkowski RK, Kaczmarek L, Popa-Wagner A (2012). Post-stroke depression: mechanics, translation and therapy. J Cell Mol Med..

[29] Popa-Wagner A, Buga AM, Tica AA, Albu CV (2014). Perfusion deficits, inflammation and aging precipitate depressive behaviour. Biogerontology..

[30] Bergdahl E, Gustavsson JM, Kallin K (2005). Depression among the oldest old: The Umeå 85+ study. Int Psychogeriatr..

[31] Skinner CJ, Holt D, Smith TMF (1989). Analysis of complex surveys.

