# Supplementary material for: The prevalence of stroke and depression and factors associated with depression in elderly people with and without stroke
Source: BMC Geriatr. 2016 Oct 7;16:174. doi: 10.1186/s12877-016-0347-6 (PMC5055663; doi:10.1186/s12877-016-0347-6)
Supplement: Additional file 1: — Survey questions. Contains the questions and answers that the present study is based on. (PDF 15 kb) [file 12877_2016_347_MOESM1_ESM.pdf]

Survey questions

"Have you had a stroke?"
Yes
No

“Are you basically satisfied with your life?”
Yes
No

“Do you feel that life is empty?”
Yes
No

“Are you afraid that something bad is going to happen?”
Yes
No

“Do you feel happy most of the time?”
Yes
No

“Do you feel depressed?”
Yes
No

“Have you had a myocardial infarction?”
Yes
No

“Have you had any kind of cancer?”
Yes
No

“Do you have diabetes?”
Yes
No

“Do you shower without human assistance?”
Yes
No

“Do you clean your residence by yourself, without human assistance?”
Yes
No

“Do you buy your own groceries without human assistance?”
Yes
No

“Do you utilize public transportation without human assistance?”
Yes
No

“Do you cook your own food without human assistance?”
Yes
No

“Are you able to read the text in a newspaper?”
Yes, without glasses
Yes, with glasse
Yes, with some other vision aids
No

“Are you able to hear what someone tells you in a normal tone of voice from a distance of about 1 meter?”
Yes
Yes, with a hearing aid
No

“Has your weight decreased in the last three months?”
Yes, more than 3 kg
Yes, between 1 and 3 kg
Don't know
No, no weight loss

“Do you live together with someone?”
Yes, with a husband/wife/partner
Yes, with a sibling
Yes, with children
Yes, with grandchildren
Yes, with another relative
Yes, with someone else, who? (free text)
No, I live alone
No, I am living with someone, but not in the same residence

“Do you have someone you can talk to about anything, someone you can share your concerns as well as your joys with?”
Yes, a husband/wife/partner/"partner living apart together"
Yes, children
Yes, grandchildren
Yes, other relative
Yes, friends
Yes, neighbors
Yes, home care services personel
Yes, a district nurse/health care professional
Yes, someone else, who? (free text)
No, no one

What is your educational background? Note the highest education level:
Less than 6 years "folkskola" (type of elementary school)
"Folkskola", "mellanskola" (9 years maximum) (type of elementary school)
Girls' school
Folk High school
Vocational school
High school diploma

University education

“Do you make ends meet?”
It is very difficult
It is pretty difficult
With some difficulty
Without any difficulty

“Have you had any aches or pain in the last week?”
Yes
No

“Have you experienced something that you would describe as a life crisis the last year (12 months)?”
Yes, personal illness
Yes, illness in relatives
Yes, death in the family, whose? (free text)
Yes, death in circle of friends
Yes, separation/divorce, whose? (free text)
Yes, other family issues, whose? (free text)
Yes, moving to another city
Yes, worsening economic state
Yes, something else, what? (free text)
No, there have been no such changes
